# Elucidating and mining the *Tulipa* and *Lilium* transcriptomes

**DOI:** 10.1007/s11103-016-0508-1

**Published:** 2016-07-07

**Authors:** Natalia M. Moreno-Pachon, Hendrika A. C. F. Leeggangers, Harm Nijveen, Edouard Severing, Henk Hilhorst, Richard G. H. Immink

**Affiliations:** 1 Physiology of Flower Bulbs, Department of Plant Physiology, Wageningen University, Wageningen, Netherlands; 2 Laboratory of Bioinformatics, Wageningen University, Wageningen, Netherlands; 3 Max Planck Institute for Plant Breeding Research, Carl-von-Linné-Weg 10, 50829 Cologne, Germany; 4 Wageningen Seed Laboratory (WSL), Laboratory of Plant Physiology, Wageningen University, Wageningen, Netherlands

**Keywords:** Tulip (*Tulip sp*), Lily (*Lilium sp*), Genes, Transcriptome browser, Sequencing, De novo assembly

## Abstract

Genome sequencing remains a challenge for species with large and complex genomes containing extensive repetitive sequences, of which the bulbous and monocotyledonous plants tulip and lily are examples. In such a case, sequencing of only the active part of the genome, represented by the transcriptome, is a good alternative to obtain information about gene content. In this study we aimed to generate a high quality transcriptome of tulip and lily and to make this data available as an open-access resource via a user-friendly web-based interface. The Illumina HiSeq 2000 platform was applied and the transcribed RNA was sequenced from a collection of different lily and tulip tissues, respectively. In order to obtain good transcriptome coverage and to facilitate effective data mining, assembly was done using different filtering parameters for clearing out contamination and noise of the RNAseq datasets. This analysis revealed limitations of commonly applied methods and parameter settings used in de novo transcriptome assembly. The final created transcriptomes are publicly available via a user friendly Transcriptome browser (http://www.bioinformatics.nl/bulbs/db/species/index). The usefulness of this resource has been exemplified by a search for all potential transcription factors in lily and tulip, with special focus on the *TCP* transcription factor family. This analysis and other quality parameters point out the quality of the transcriptomes, which can serve as a basis for further genomics studies in lily, tulip, and bulbous plants in general.

**Electronic supplementary material:**

The online version of this article (doi:10.1007/s11103-016-0508-1) contains supplementary material, which is available to authorized users.

## Introduction

Modern sequencing technology, also referred to as next generation sequencing (NGS), quickly generates large amounts of sequence data at lower cost in comparison with traditional Sanger sequencing (Marguerat and Bähler 2010; Schatz et al. 2010). While sequencing and assembly of large genomes still represent a technical challenge and a laborious procedure (Treangen and Salzberg 2011), sequencing the expressed part of the genome, represented by the transcriptome, is nowadays achievable and can level down the complexity and provide useful information (Riesgo et al. 2012). Therefore, transcriptome sequencing may represent an alternative to whole genome sequencing for species with large complex genomes when the aim is to generate a comprehensive database of genomic resources, suitable for gene identification, allele mining, or genome wide expression studies (Duangjit et al. 2013; Hou et al. 2011; Liu et al. 2012).

**Fig. 1:**
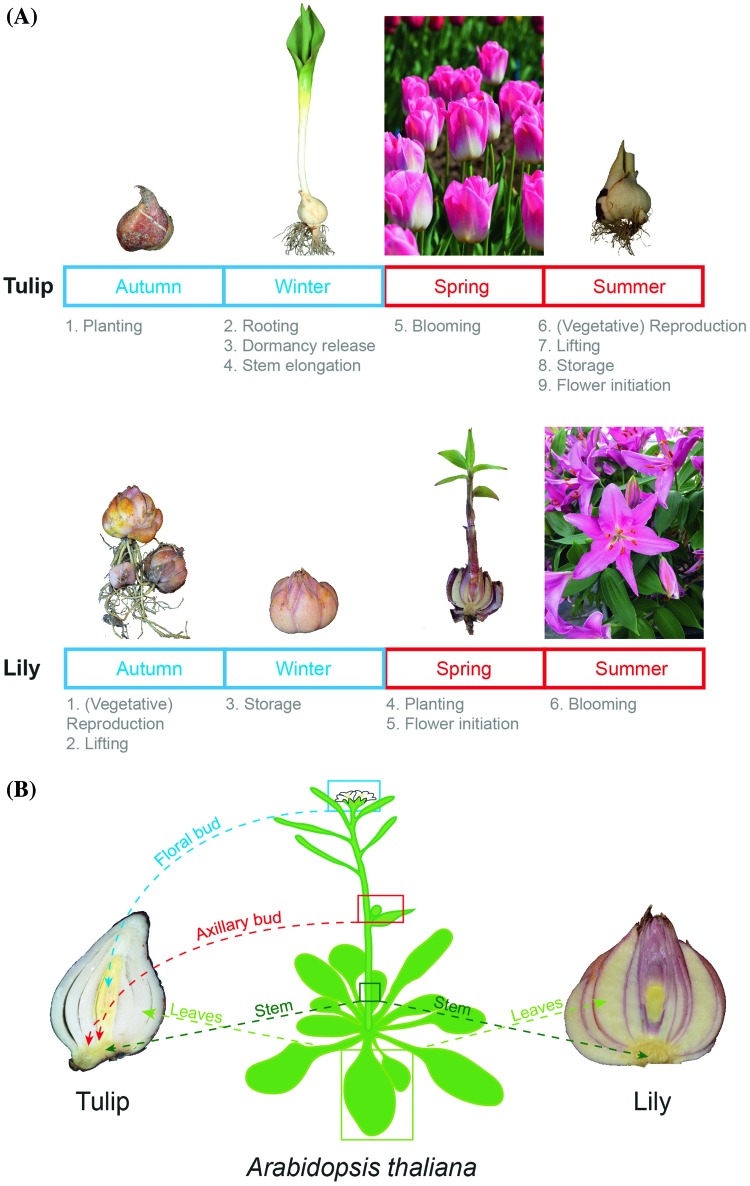
Life cycle and architecture of tulip and lily bulbs. **a** Tulip and lily yearly growth cycle. Note that their growth cycle is very similar. Both require a period of cold, but for different purposes and blooming occurs in different seasons. **b** Bulbs can be regarded as modified plants where the stem has shorten into a basal plate, the leaves have been modified into bulb-scales. In the tulip bulb the axillary buds are located in the axils of the bulb-scales and the floral bud is located in the center on top of the basal plate

Bulbous plants, also classified as geophytes, represent species with economic relevance, large genomes and relatively scarce genomic resources. In short, geophytes are plants with storage organs and renewal buds resting in underground structures (Kamenetsky and Okubo 2013) (Fig. 1). Tulip and lily (*Tulipa sp* and *Lilium sp)* are ornamental geophytes with an estimated genome size of 25 and 36 GB, respectively (2012). One of the first studies of a transcriptome characterization for both species was done by Shahin et al. in 2012 using 454 pyro-sequencing technology of messenger RNA (mRNA) from leaves (2012). They obtained 81,791 unigenes for tulip with an average length of 514 bp and 52,172 unigenes for lily with an average length of 555 bp. Later studies have e.g. focused on sequencing the transcriptome of leaves (Wang et al. 2014), bulblets (Li et al. 2014) and meristem-enriched tissue (Villacorta-Martin et al. 2015) of different *Lilium* cultivars, using the Illumina HiSeq sequencing platform. These studies resulted in the identification of 37,843 unigenes for leaves (Wang et al. 2014), 52,901 unigenes in bulblets (Li et al. 2014) and 42,430 genes for the meristem-enriched lily tissues (Villacorta-Martin et al. 2015).

Despite continuous efforts to broaden the genetic resources of the bulbous species tulip and lily, characterization of their entire transcriptome is far from being completed. The information generated to date only covered leaf and meristem-enriched tissues and, furthermore, the data is difficult to access and mine for non-bioinformaticians. Our study aimed to generate a high quality and extensive transcriptome of these two bulbous species and making this valuable resource publicly available through a user-friendly and freely accessible web-based interphase, allowing easy data mining. The Illumina HiSeq platform was used to sequence a pooled sample for lily and for tulip, each made up of a mixture of equal amounts of poly adenylated mRNA obtained from flowers, stem, leaves, bulb and bulblets. Even though short reads are generated with the Illumina HiSeq platform, a tremendous throughput can be reached, resulting in an improved coverage of rare transcripts in comparison to the other platforms used in some of the previous transcriptome studies of bulbous species (Kamenetsky et al. 2015; Shahin et al. 2012).

The generated data was used to assemble reference transcriptomes for tulip and lily. For this purpose, different assembly settings were explored, aiming to generate an optimal transcriptome for gene mining. To proof the quality of the generated data sets, a comparison was made between the transcripts found in the bulbous species tulip and lily and the genes of the model species *Arabidopsis thaliana* and *Oryza sativa* (rice). In addition, we searched for potential transcription factors present in both transcriptomes and compared their distribution with the distribution of transcription factors in the model species Arabidopsis and *O. sativa*. Subsequently, a web-based interface (Kamei et al. 2016), which we call Transcriptome Browser, was implemented for data presentation and mining. The various possibilities of this browser are exemplified by zooming-in on a particular plant-specific gene family and the identification of all potential members of this transcription factor family. This activity enlightens the usefulness of the tulip and lily transcriptome browser in mining high-throughput sequencing data and identifying sequence information from lowly expressed, but important regulatory genes. Furthermore, these analyses revealed the quality of our data set and show how this resource can be explored in the future to study biological processes in bulbous plants at the molecular level.

## Results

### Transcriptome sequencing and assembly

The Illumina HiSeq 2000 platform was used to sequence the tulip and lily transcriptome of a wide range of tissues varying from bulb scales to flowers. After trimming and removal of low quality reads, a similar number of paired end reads were obtained for both libraries: 169,920,574 reads for tulip and 165,031,389 for lily. Subsequently, Trinity software (Grabherr et al. 2011) was used to assemble both transcriptomes de novo and this assembly yielded to 499,780 transcripts for tulip and 569,305 for lily with an average length of 561 and 487 bp, respectively. When not taking the isoforms into account and without applying additional data filtering, Trinity predicted 380,091 genes for tulip and 467,241 for lily (Table 1). Transcript over-estimation is common in de novo sequencing studies because the lack of a reference transcriptome or genome limits the assembly of sequences that represent non-overlapping pieces of the same gene. Transcripts expressed at extremely low levels can also cause noise because they may not be reliably assembled (http://cole-trapnell-lab.github.io/cufflinks/cufflinks/). Furthermore, it is difficult to distinguish between isoforms of one gene versus the existence of more gene copies as a consequence of duplications (Chang et al. 2015).

**Table 1:** Summary statistics of the tulip and lily transcriptomes generated by non-filtered data and upon applying three different filtering parameter settings

	Non-filtered		Counts per transcript ≥10		Counts per transcript ≥20		TPM ≥1	
	Tulip	Lily	Tulip	Lily	Tulip	Lily	Tulip	Lily
Contigs	499,780	569,305	174,442	252,040	112,256	131,912	39,171	38,688
Genes	380,091	467,241	115,167	198,613	70,634	94,283	29,523	29,188
GC %	42.74	41.79	43.62	42.1	43.98	42.64	45.4	45
N50	695	514	1226	913	1478	1322	1573	1717
Average length	561	487	933	703	1.139	989	1.017	1.035

*TPM* transcripts per million

Therefore, filtering out lowly expressed transcripts is a routine procedure applied during transcriptome assembly to get rid of noise and contamination, and it yields, in general, significantly reduced numbers of predicted transcripts and genes. To compare and find the optimal parameters for our two datasets, but retaining the full complexity of the tulip and lily transcriptomes, we generated three additional assemblies based on different abundance filtering settings. The three new assemblies consisted of transcripts with equal or more than 10 or 20 counts; and transcripts occurring at least more than once per million (TPM), respectively. As summarized in Table 1, increasing the cut-off value to filter out transcripts with low abundance leads to a dramatic decrease in the number of predicted transcripts and genes, but improves the N50 and average transcript length.

The number of obtained transcripts and predicted genes, in combination with the average transcript length, is generally used as a quality indicator of de novo transcriptome assemblies. In an ideal situation, the number of predicted genes should be close to the number of genes expected for the species. Based on this criterion, using counts per transcript upward of 20, seemed to be the best parameter since it reached a reasonable number of genes taking into account the number of genes found in sequenced plant genomes [e.g. rice (International Rice Genome Sequencing Project 2005); Arabidopsis (The Arabidopsis Genome Initiative 2000); poplar (Tuskan et al. 2006); loblolly pine (Neale et al. 2014)]. Furthermore, this filtering resulted in a high average transcript length, suggesting a high percentage of complete and fully covered mRNA sequences in this assembly.

**Fig. 2:**
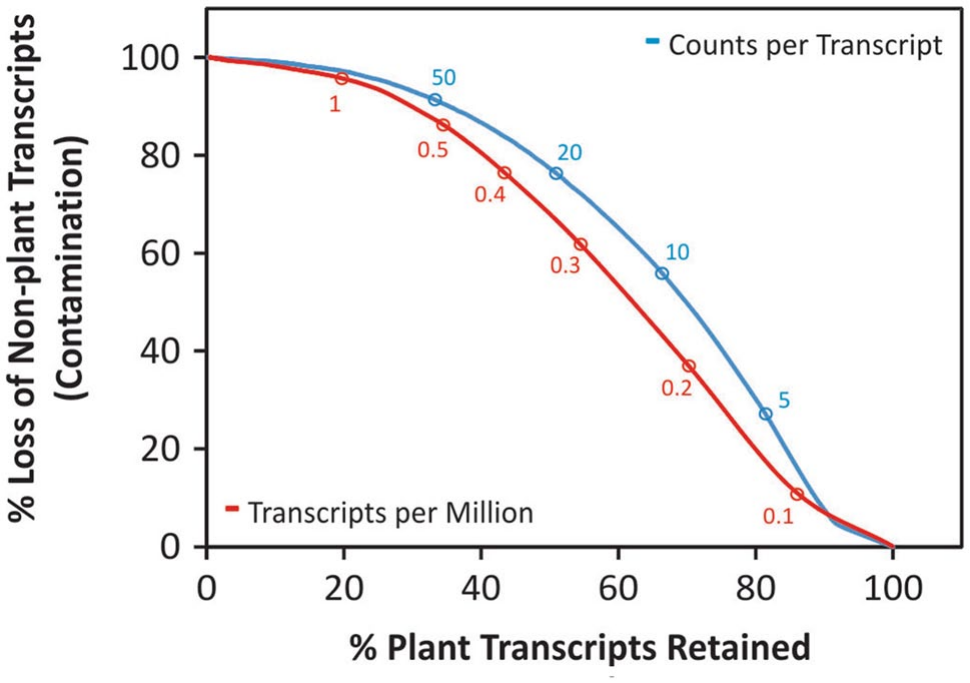
Effect of transcriptome filtering on the percentage of “non-plant” and plant transcripts retained. Filtering done based on counts per transcript and transcripts per million

Nonetheless, it is important to realize that the high number of transcripts and predicted genes in the non-filtered transcriptome may not only be the result of miss-assemblies and non-plant contamination, but also because of the presence of incomplete or truncated rare, but valuable transcripts. Such incomplete transcripts may be the result of incomplete cDNA amplification, or mRNA degradation and breakage, and in general lowly expressed transcripts are more prone to be assembled as fragments due to limited sequencing coverage. To investigate this option in more detail, we studied – using the lily transcriptome as an example – how filtering out lowly expressed transcripts affects the number of transcripts encoding plant orthologues as well as the transcripts considered to be contamination (Fig. 2). As expected, the three filtering options improved the raw transcriptome in terms of contamination, but surprisingly decreased also dramatically the number of plant orthologues retained. For example, TPM larger or equal to one reduced the contamination with almost 100 % efficiency, but only retained a bit more than 20 % of the plant orthologues from the non-filtered transcriptome database.

This observation prompted us to gain more insight in the nature of the transcripts with low abundance. For this purpose, all removed transcripts per filtering method were compared with the Arabidopsis information resource (TAIR) database using the Basic Local Alignment Search Tool (BLAST). Within the removed transcript sequences many important gene products where present, e.g. encoding putative meristem signalling peptides (CLAVATA3/ESR) (Wang and Fiers 2010), which are known to be short in sequence and lowly expressed. Furthermore, transcript fragments of genes expected to be very locally and lowly expressed, such as some basic helix-loop-helix (bHLH) transcription factors, wound-responsive protein-related and flowering promoting factors, were identified in these filtered-out transcript sets (see Supplemental Table 1). Hence, the use of a filtering method may lead to a transcriptome with improved quality based on average transcript length, but, it results on the other hand in the removal of a substantial number of transcript fragments corresponding to important plant genes. Based on these observations, we decided to continue with a non-filtered transcriptome, including short, truncated, and incomplete transcripts, since this increases the chances of identifying sequence information of rarely expressed genes. In order to evaluate the completeness of these final assembled and selected tulip and lily non-filtered transcriptomes, core eukaryotic genes mapping approach (CEGMA) analysis was used (Parra et al. 2007), showing that the generated transcriptomes of tulip and lily contain nearly 100 % of the 248 core eukaryotic proteins (98.79 % for both species).

### Functional annotation

TransDecoder 2.0.1 (Haas et al. 2013) has been used to predict coding sequences in the tulip and lily transcriptomes. Subsequently, the UniProt protein database (Consortium TU 2015) and the Pfam conserved domain database (Finn et al. 2014) were used to predict protein coding genes. In total 147,101 transcripts of tulip were identified, resulting into 89,530 predicted protein coding genes and 144,801 transcripts of lily, giving rise to 101,312 predicted genes. Those predicted genes represent nearly 50 % of the transcripts in the non-filtered transcriptomes. In a follow-up step, the predicted proteins of tulip and lily were grouped in so-called orthology clusters (oc) using OrthoFinder (Emms and Kelly 2015). The clusters also contained the monocots rice, maize, Brachypodium, sorghum, switchgrass, barley and garlic; and the dicots soybean, Arabidopsis, grape, poplar and tomato. A total of 15,296 orthology groups were found to contain lily and tulip proteins, 10,014 of these also included one or more Arabidopsis proteins. A search for orthology groups that only contained proteins from the bulbous species tulip, lily, and garlic (Kamenetsky et al. 2015), revealed a set of 281 unique groups that might represent bulbous plant specific genes.

To get a better impression of the quality and completeness of the functional annotated datasets, we compared our transcriptomes and annotation with previously published transcriptomes of tulip and lily Shahin et al. (2012). Initially, we performed a BLAST search at the nucleotide level to determine how well we covered the transcripts present in these publicly available datasets. Depending on the cultivar we used for this comparison, we found a BLAST hit for 87–95 % of the published tulip contigs and for 80–85 % of the lily contigs. These numbers reveal that we found evidence for the presence of the majority of potential genes in the published datasets in our transcriptomes. Subsequently, we determined how many potential tulip and lily genes with a putative Arabidopsis ortholog were unique in either our transcriptomes, or the published datasets of Shahin et al. (2012). For this purpose a BLAST screening (blastx, e-value cut-off of 1e-5) on the Arabidopsis proteome was performed for the individual datasets. In this analysis we found 1345 and 95 unique tulip hits, for the transcriptomes described in this study and the published tulip datasets, respectively. For lily these numbers were 647 and 164. So on average almost eight times more additional and unique sequences with a BLAST hit to the Arabidopsis proteome were identified in this study in comparison to the previous study. In Supplemental Table 2, an overview is presented of the unique hits in the individual lily datasets as an example. As expected, a large part of the unique sequences in our transcriptomes in comparison to the published transcriptomes resemble genes that are expressed in tissues other than leaves, which was the only tissue sampled by Shahin et al. (2012). In addition, sequences were uniquely identified in this study that are potentially encoding for rare and low expressed genes. Examples are three out of 22 known members of the novel seed plant-specific family of small peptides encoding genes, *ROT-FOUR LIKE1-22 (RTFL1-22)* (Narita et al. 2004).

### Transcriptome coverage assessed by the identification of transcription factor families

**Fig. 3:**
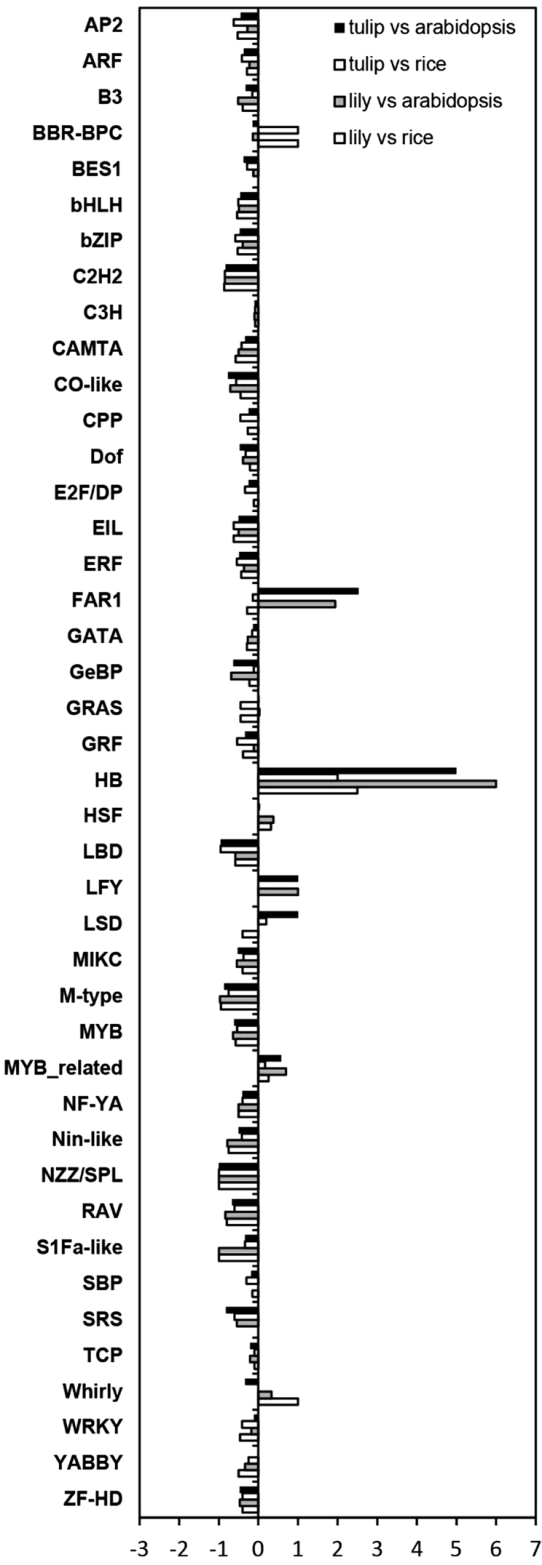
Overview of 42 transcription factor families identified in lily and tulip in comparison to rice and Arabidopsis. The *bar* represents the relative number of transcription factors present in each family in comparison to the number of transcription factors present in the model species Arabidopsis and rice, respectively. A value below one indicates under-representation in lily or tulip in comparison to rice or Arabidopsis and a value above one shows over-representation

In the plant kingdom a large number of transcription factor families can be found and they are involved in several processes, ranging from plant development to abiotic and biotic stress responses (Riechmann et al. 2000; Zhang et al. 2011). Transcription factors orchestrate several networks by controlling when and where certain genes will be expressed (Lee et al. 2006) and, therefore, have been well studied and characterized in plants. However, even though they function as master regulators, transcription factors are often expressed at relatively low abundance (Jones et al. 2015). This low level of expression makes transcription factors suitable markers to further assess the sequencing depth and coverage of our two generated transcriptomes. Therefore, a comparison was made between the 42 known transcription factor families in the model species Arabidopsis and rice, and our generated transcriptomes of tulip and lily. For this purpose, the putative transcription factors of each family were identified based on Pfam domains (Finn et al. 2014). The outcome of this analysis is summarized in Table S2. A large number of transcription factors were identified in the transcriptome data of both lily and tulip with an expected distribution over families, but some families in both tulip and lily seemed to contain more putative members than expected based on their abundance in model species (Fig. 3). Examples are the homeodomain (HB) family and the MYB related transcription factor family. For the FAR1 family, over-representation is observed in comparison to Arabidopsis but the numbers found in lily and tulip, are almost equal in comparison to rice. This might point to a monocot specific expansion of this specific transcription factor family. In general, the number of transcription factor members in a particular family is rather similar in the two bulbous plant species. However, exceptions can be found for the zinc finger LSD and the Whirly family. The LSD family is over-represented in tulip while the Whirly family is over-represented in lily, based on our datasets. These examples might point to species-specific family expansions, though additional analyses are needed before firm conclusions can be drawn.

**Fig. 4:**
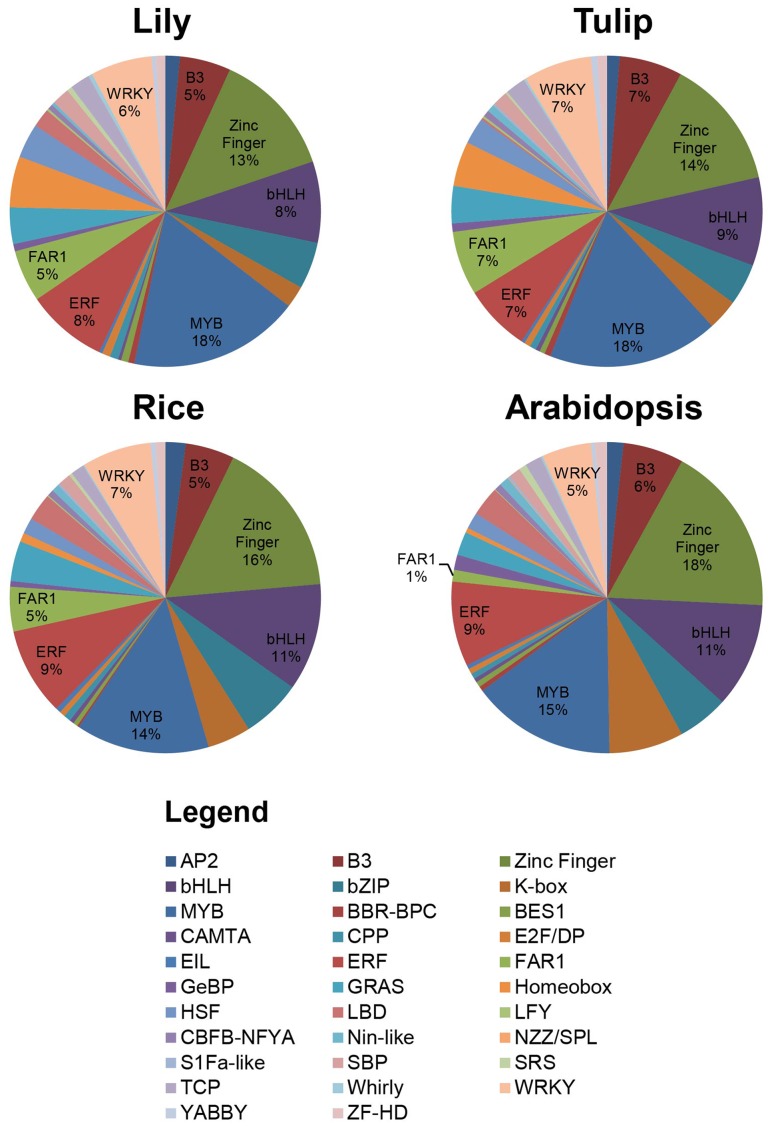
Distribution of transcription factors based on conserved protein domains in lily, tulip, Arabidopsis and rice. The transcription factor family distribution in tulip and lily is similar to the distribution in rice and Arabidopsis. However, in comparison with Arabidopsis, the FAR1 transcription factor family is larger in the monocots tulip, lily, and rice

A further in depth analysis was made by focussing on the presence of characteristic transcription factor protein domains and comparing them among plant species. In this respect it is good to realize that several families contain a common protein domain and that due to fragmentation of the obtained transcriptomes it may be difficult to distinguish these transcription factor families into sub-classes. Examples are the M-type and MIKC MADS domain transcription factor family clades, AP2 and RAV, B3 and ARF, and HB-other and HB-PHD (Riechmann et al. 2000). In Fig. 4 an overview is given of the distribution of TF protein domains within each species. As expected, the overall distribution is similar between the model species and the bulbous plants tulip and lily. One of the largest groups of transcription factors, which covers ~13–15 % of all transcription factors of the 42 families, contains a zinc finger domain. The second largest group is represented by the MYB transcription factors (~12–15 %), followed by the bHLH domain containing transcription factors (~7–10 %). A major and remarkable difference is observed between monocotyledonous and dicotyledonous species for the FAR1 domain containing transcription factors, as was already mentioned above. Approximately 5–6 % of the total transcription factors used in this analysis has the FAR1 domain in lily, tulip and rice. Nevertheless, in Arabidopsis only ~1 % of the transcription factors contain this domain. The biological relevance of the expansion of this particular transcription factor family in tulip and lily is currently not known, but it seems not to be an assembly artefact, since the overrepresentation is also found in the completely sequenced rice genome (International Rice Genome Sequencing Project 2005).

### Mining high throughput data with the *transcriptome browser*: identification of the TCP gene family

**Fig. 5:**
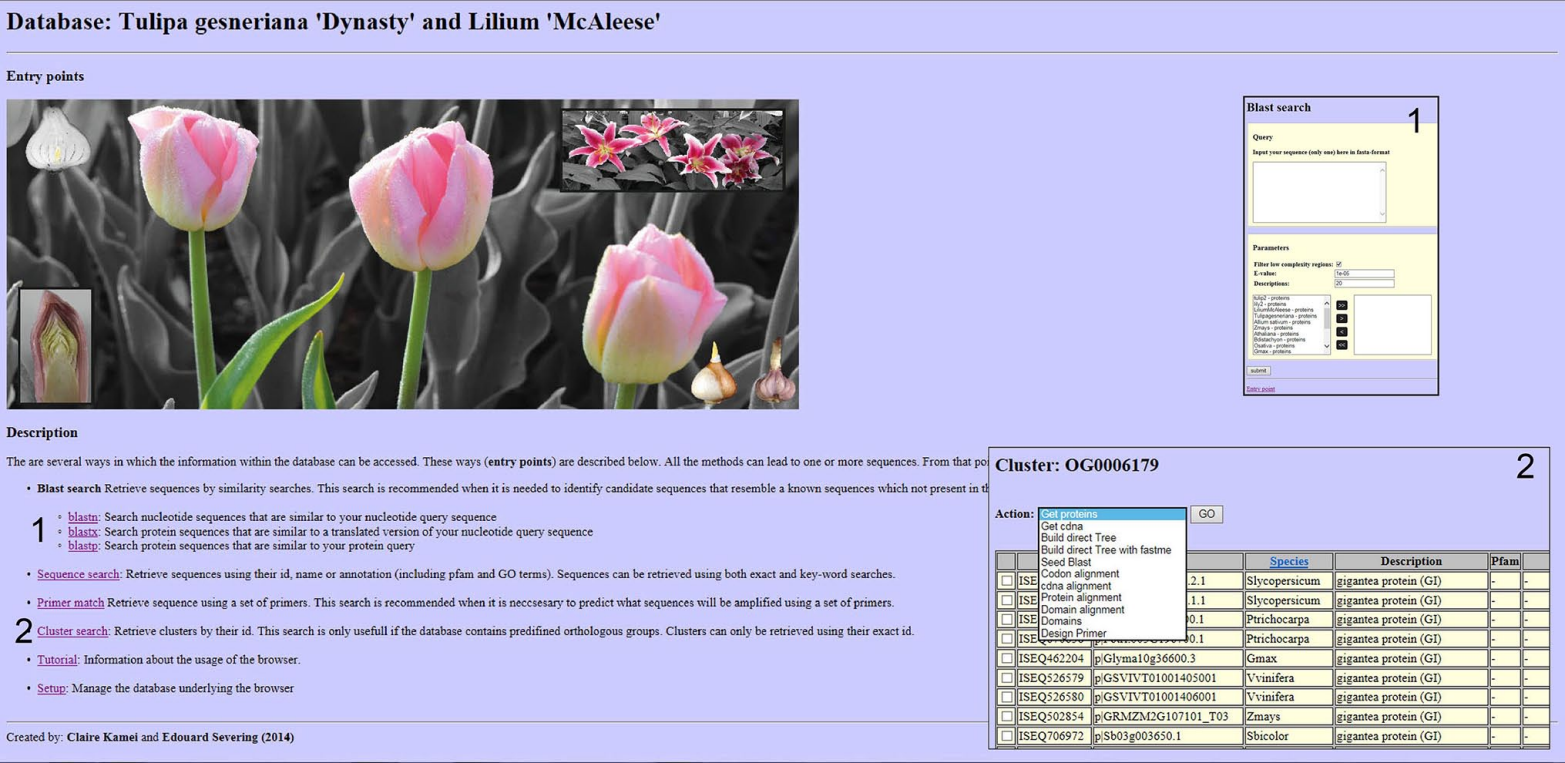
Screenshots of the Transcriptome browser. In *panel 1* the interphase of the BLAST search is shown. The input sequence can be blasted against the tulip and lily transcriptomes as well as other plants species such as *Arabidopsis thaliana, Oryza sativa* and *Vitis vinifera*. In *panel 2* an example is given of the output of the cluster search. Here different actions can be chosen such as protein alignment, primer design and build a direct tree (phylogenetic tree). Note that the browser has a tutorial option, in which the exact procedure how to perform the different tasks and actions is explained

Once a transcriptome is assembled, one of the biggest challenges for researchers is to explore the large dataset in search for sequences with biological relevance. To support in mining data using open sources, we decided to deposit our generated transcriptomes in a web-browser (http://www.bioinformatics.nl/bulbs/db/species/index) based on recently developed open software (Kamei et al. 2016). This web-based interface offers basic bioinformatics search tools, identification of candidate transcripts based on phylogenetic relationships between orthologous sequence data and design of specific and degenerate primers for expression studies of transcripts of interest (Fig. 5).

To explore the usefulness of this data resource, we mined the datasets aiming to identify members of the *TCP* gene family in lily and tulip. The *TCP* transcription factor family, named after its founder members *TEOSINTE BRANCHED1, CYCLOIDEA*, and *PROLIFERATING CELL FACTOR*, has in general around 25–30 members in eudicots (Nicolas et al. 2015). *TCP* genes are expressed in a wide range of tissues and they control flower, leaf, and lateral shoot growth by activating or inhibiting cell proliferation (Martín-Trillo and Cubas 2010; Nicolas et al. 2015; Mondragón-Palomino and Trontin 2011). Furthermore, evidence from Arabidopsis expression studies indicates that several *TCP* members are lowly expressed in the above ground tissues (Danisman et al. 2013).

The expected wide-range in tissue and level of expression of *TCP* genes was our reason to choose this gene family to assess the power of the *Transcriptome Browser* in mining high throughput sequencing data. All putative lily and tulip *TCP* sequences were identified by using the sequence search tool (setting Pfam PF03634), followed by seed BLAST analyses with different parameter settings, and an additional manual search scrolling through the orthology clusters (oc). The Pfam search resulted in 38 tulip and 33 lily transcripts, the seed BLAST search into two additional tulip transcripts and the oc search identified two extra transcripts for each species. This total of 42 tulip and 35 lily transcripts, represented 24 and 22 potential *TCP* genes respectively.

**Fig. 6:**
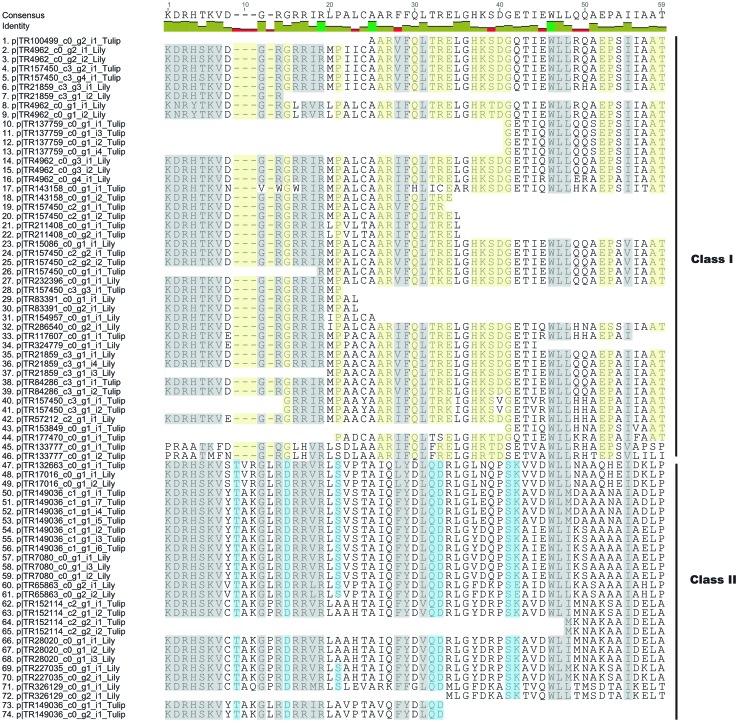
Sequence alignment of the domain of 74 *TCP* transcripts found in tulip and lily. Sequences are clustered in class I and class II based on the classification by Martín-Trillo and Cubas (2010). Sequences 64 (p|TR152114_c2_g2_i1_Tulip) and 65 (p|TR152114_c2_g2_i2_Tulip) were found by seed BLAST search only, and sequence 7 (p|TR21859_c3_g1_i2_Lily) was identified by the orthology cluster (oc) search option. *Yellow* shaded regions indicate characteristic features of class I, *blue* characteristic features for class II and *grey* conserved amino acids in both classes

The following step was to corroborate the TCP identity of the resulting tulip and lily transcripts based on the characteristic features of the TCP domain described by Martín-Trillo and Cubas (2010). As shown in Fig. 6, the two putative *TCP* transcripts identified by seed BLAST search, as well as the remaining lily transcript found by oc search contained only a partial fragment of the TCP domain and this was the reason why they failed to pop-up within the PFAM search. However they can be considered true TCPs based on their characteristic features. This example shows the power of using the *Transcriptome Browser* in data mining and highlights the importance of our choice to maintain truncated transcripts into the final assembly.

Although the aim of this study was not to characterize the identity of each *TCP* transcript found in tulip and lily, we wanted to test the capacity of the *Transcriptome Browser* in clustering the tulip, lily, rice and Arabidopsis TCP sequences, based on sequence similarity. All lily, tulip, Arabidopsis and rice protein sequences which contained the TCP domain (from the initial Pfam search) were selected to build an unrooted tree using the Neighbour-Joining algorithm (Fig. S1). Once again, the browser was able to distinguish between transcripts from class I and II. Also, most of the clades contained transcripts of all four species, which might help in further approaches to characterize the TCP identity of the tulip and lily transcripts.

Last, we tested the capacity of the “specific primer design tool” offered in the *Transcriptome Browser* (Kamei et al. 2016). This tool designs primers in unique regions, given a set of similar sequences. PCR amplification of unspecific fragments or fragments without the expected size might indicate assembly errors. Therefore, five *TCP* genes were selected randomly for each bulbous species. The browser was able to design unique primers in all chosen sequences and PCR amplification with the expected band size was observed in nine out of the ten selected genes (Fig. S2). Overall, this result highlights the power of the de *Transcriptome Browser* in designing specific and unique primers given from e.g. the members of a gene family.

## Discussion

Despite various large-scale sequencing efforts, we still lack a comprehensive transcriptome for many species. In this study a large-scale lily and tulip transcriptome was generated and this resource has been made available in a web-browser for easy mining.

### Filtering out transcripts with low abundance reduced the number of retained plant orthologue hits

The number of transcripts and predicted genes in our non-filtered transcriptomes may be highly over-estimated taking into account that there are only 27,024 protein coding gene models in the recently sequenced monocot genome of pineapple (Ming et al. 2015), 39,045 genes reported for rice––the monocot model species––(International Rice Genome Sequencing Project 2005) and 81,791 for tulip, based on a previous transcriptome sequencing effort Shahin et al. (2012). Nevertheless, we expect that the large number is certainly not all because of noise, whereas the methodology we selected for sequencing assures high depth coverage and strand specificity. These aspects make the identification of rare and lowly expressed transcripts for both coding and non-coding RNAs possible. Additionally, both tulip and lily are in general vegetative propagated and therefore heterozygosity is maintained, being a source for a higher number of different transcripts. Although in other bulbous studies filtering out low abundant sequences reduced significantly the number of predicted genes to a level that gets close to what is reported for model species (Villacorta-Martin et al. 2015), we proved that in our data this filtering reduced dramatically the percentage of transcripts with substantial homology to a known plant gene. Therefore, our non-filtered transcriptomes may not reflect the true number of genes but they rather represent extensive transcriptome coverage for both tulip and lily. Despite the fact that there is some contamination (non-plant hits) retained in the non-filtered databases, both transcriptomes contained nearly 100 % of a core set of eukaryotic proteins, which is an indication of the completeness of the assemblies. Furthermore, we showed the power of these transcriptomes in finding rarely expressed genes, such as genes belonging to the *CLV*/*ESR* family encoding for small size ligands that act as important developmental signalling molecules (Wang and Fiers 2010).

### Transcriptome coverage assessment

In addition to the core eukaryotic proteins, the transcription factor family distribution analysis in tulip and lily has also confirmed the quality of the transcriptome assembly. A large number of transcription factors could be identified even though not all tissues, developmental stages, and common biological process––such as stress responses and floral primordium formation-of the bulbs were collected for RNA-sequencing. To mention an example, tulip tissues were collected from January until May, leaving out the months June to December. During this latter period of time, the floral primordium inside the tulip bulbs is formed (Khodorova and Boitel-Conti 2013) and therefore transcription factors specifically involved in this process might be absent. When zooming in on the members of each transcription factor family found in tulip and lily, some families contain more members in comparison to the model species or vice versa. Although, we cannot rule out miss-assembly as a reason for over-representation in particular transcription factor families, a few nice examples of expanded families have been found that based on comparison with other monocots seem to be present and probably unique to monocots or bulbous species. Having more members in a family can be due to the large genome that both tulip and lily have which might be partially due to additional gene duplication events. It will be of interest to study in the future whether there are bulbous-plant-specific functions for these additional genes, proving their biological relevance. Though, before going into laborious in-depth functional studies it is essential to confirm a correct assembly of these potential novel genes by wet-lab experiments, other sequencing methods such as PacBio, or using software such as recognition of errors in assemblies using paired reads (REAPR) (Hunt et al. 2013).

### Functionality of the transcriptome browser in mining the extensive tulip and lily transcriptomes

Mining high through-put data often requires advanced programming skills or access to user friendly commercial software. Most of the publicly available software tools offer limited options, forcing researchers to use a combination of open software packages, requiring in general different formats and operational systems (Deng 2011). Based on the identification of the putative *TCP* transcripts for both bulbous species, we confirmed that the *Transcriptome Browser* (Kamei et al. 2016) represents a reliable and user-friendly web based interface, able to identify gene families and build phylogenetic relationships with other species.

## Conclusion

The methodology implemented in this study to assemble de novo transcriptomes demonstrates that there is a trade-off between transcriptome quality and the amount of information retained. Filtering out data that are considered “noise” improves the values of the parameters that are commonly used to assess the quality of a transcriptome. However, such filtering methods may limit the power of data mining by e.g. reducing dramatically the chances of finding rare or lowly expressed genes. This study resulted in extensive transcriptome resources for both tulip and lily that can be easily mined. The limited number of molecular studies performed in these two bulbous species to date, states the need for such a user-friendly resource. Although, genome sequencing has undergone an enormous revolution over the last decade, it will most likely take some time before a high-quality and well-assembled genome sequence of lily and tulip will become available. Until that moment, the transcriptome browser presented here will be of pivotal importance for gene identification in these two bulbous plant species.

## Methods

### Plant material

Tulip and lily tissues of several developmental stages were collected throughout the year of 2013 in The Netherlands. Adult tulip bulbs of the cultivar “Dynasty” (*Tulipa gesneriana*) were planted in October 2012 in the field at Wageningen University (51.9667°N, 5.6667°E). Tulip bulb-scales, axillary buds, stem, leaves and floral bud were collected in January when all organs were entirely below ground; in March when the stem and leaves emerged above ground; and in May during blooming at full anthesis of the flowers. Roots and just initiated and dormant flower buds inside the buds during summer have not been sampled.

Tissues of lily cultivar “McAleese” (*Lilium*, oriental hybrid group) were collected from regenerated bulblets and from fully grown plants. Regenerated bulblets were obtained by incubating detached bulb-scales in moist chambers without exogenous hormonal application at 23 °C for 6 weeks, followed by 12 weeks at 4 °C. Newly regenerated bulblets were dissected under a stereo microscope and collected at the developmental stages S0 (proximal side of the explant at the start of the culture); S1 (proximal side of the explant at 1 day after culture); S2 (thickened structures of proximal side of the explant); D (dome formation); P (bulb-scale primordium formation); B (bulblet formation) (Marinangeli et al. 2003). Fully formed regenerated bulblets were also collected at 6 weeks after culture under 23 °C (bulblets are thought to enter a resting phase at this moment); and at 18 weeks after culture, from which the first 6 weeks were at 23 °C followed by 12 weeks at 4 °C (bulblets are out of the resting phase and ready to sprout into leaflets or a true stem). In addition to the regenerated bulblets, fully grown leaves, closed and open flowers, stem, and stem axils containing axillary buds were collected at the moment of blooming from greenhouse-grown plants (In the Netherlands; Long day (~16 h of light) conditions and 20–25 °C). After collection of both tulip and lily plant material, the tissues were ground in liquid nitrogen and stored in −80 °C until use.

### RNA isolation

Total RNA was extracted from tulip bulb-scale tissue with the Tripure protocol (Roche, The Netherlands) according to the manufacturer’s manual, with the addition of 2 % Polyvinylpyrrolidone (PVP, w/v) and 2 % β-mercaptoethanol (β-ME, v/v) to the extraction buffer. Isolated RNA was DNase treated with RQ1 (Promega, The Netherlands) followed by a phenol/chloroform (1:1) extraction and ethanol precipitation. RNA from the other tulip tissues was extracted with the Invitrap spin plant RNA mini kit (Invitek, ISOGEN Life Science, The Netherlands) and DNase treated with DNaseI (Qiagen, The Netherlands).

Total RNA from all tissues collected from lily plants was isolated following the Tripure protocol (Roche, The Netherlands) with modifications. The modifications consisted of an initial removal of starch using an SDS-containing buffer [buffer I, (Li and Trick 2005)] followed by phenol/chloroform extraction; and a final RNA purification of the eluted pellet using the Invitrap spin column (Invitrap spin plant RNA mini kit, Invitek, ISOGEN Life Science, The Netherlands). DNA was removed from the samples by DNAse treatment with RQ1 (Promega, The Netherlands) according to the manufacturer’s specification.

Quantity and quality of isolated RNA was assessed by agarose gel electrophoresis and NanoDrop spectrophotometer ND1000. Samples with a 260 to 280 ratio ranging from 1.7 to 2.1 were selected and mixed into equal RNA quantities into a separated lily and tulip pool. These two pooled RNA samples were sent to Wageningen UR Greenomics (Wageningen, The Netherlands) for cDNA library preparation and subsequent sequencing.

### cDNA library preparation and sequencing

A cDNA library for each pooled sample was prepared following the TruSeq Stranded Total RNA Sample Preparation kit with Ribo-Zero Plant (Illumina, The Netherlands). The Ribo-Zero Plant kit removes ribosomal RNA (rRNA) from total RNA using biotinylated probes and the obtained rRNA-depleted RNA is first and second cDNA transcribed keeping strand specificity. Quality and quantity of each library was checked using a Bioanalyzer 2100 DNA1000 chip (Agilent technologies) and Qubit quantitation platform using Quant-iT PicoGreen (Invitrogen, Life Technologies). Library sequencing was done on a HiSeq2000 platform. The tulip and lily transcriptomes raw data were submitted to The National Center for Biotechnology Information (NCBI) under the numbers SRR3105600 (tulip) and SRR3105700 (lily).

### Sequencing analysis

Paired-end reads were sequenced using Illumina Hiseq 2000. The quality of the reads was examined by FastQC (http://www.bioinformatics.babraham.ac.uk/projects/fastqc/). Adapters were removed and paired-end reads were trimmed using Trimmomatic (Bolger et al. 2014) with settings: “ILLUMINACLIP:TruSeq3-PE-2.fa:2:30:10 LEADING:20 TRAILING:20 SLIDINGWINDOW:4:20 MINLEN:70 HEADCROP:5”.

The transcriptomes were assembled de novo using Trinity version 2.0.6 (Haas et al. 2013) with default settings, except max_memory 150G and SS_lib_type RF. Transcriptome statistics were determined using the TrinityStats.pl script, which is part of the Trinity package. Transcripts abundances were quantified using RSEM version 1.2.22 (Li and Dewey 2011) with default settings.

To assess the level of contamination contained in both assemblies, NCBI’s non-redundant protein database (nr) was searched using Diamond (Buchfink et al. 2015) with default settings and the results were analysed using MEGAN (Huson et al. 2007). CEGMA analysis (Parra et al. 2007) was used as a rough measure of the completeness and quality of the assemblies.

Coding sequences on the transcripts were predicted using TransDecoder version 2.0.1 (Haas et al. 2013) as follows: first the longest open reading frames (ORF) were determined and translated using a cut off of 60 amino acids as the minimal protein length. The resulting protein sequences were used as queries to search the SwissProt section of the UniProt protein database (Consortium TU 2015) with blastp (E-value cut-off 1*e-5), and they were also scanned for conserved protein domains from the Pfam (Finn et al. 2014) database using Pfamscan. The Blast hits and Pfam results were used as input for the TransDecoder.Predict tool. Subsequently, the longest peptides per transcript on the (+) strand were selected using a custom Python script.

Translated sequences were clustered with orthologous proteins from the monocots rice, maize, Brachypodium, sorghum, switchgrass, barley and the dicots soybean, Arabidopsis, grape, poplar and tomato using OrthoFinder (Emms and Kelly 2015).

### Search transcription factor families

For the identification of transcription factor families a PFAM analysis was performed on all the proteins present in the transcriptome from both lily and tulip. The families were divided according to the family assignment rules used in the Plant Transcription Factor Database (http://planttfdb.cbi.pku.edu.cn/help_famschema.php). Transcription factor families without a Pfam domain were identified with BLAST by using the known Arabidopsis thaliana transcription factors in a particular family.

### Tulip and lily transcriptome mining

Tulip and lily putative *TCP* transcripts were retrieved using the *Transcriptome Browser* in three successive steps. The first screen was achieved making use of the sequence search tool, option Pfam (PF03634). In the second step, new *TCP* transcripts were identified by selecting all tulip and lily transcripts from the first screen and using the “Seed BLAST” tool without default parameters. In the last step every oc cluster containing tulip, lily, Arabidopsis and rice transcripts with a PF03634 hit were screened manually. The TCP domain sequence of each transcript was retrieved manually from the *Transcriptome Browser* and aligned using Geneious software (Drummond et al. 2010). All Arabidopsis, rice, lily and tulip transcripts resulting from the Pfam (PF03634) search were clustered using the Neighbour-joining tree option of the *Transcriptome Browser*. Primer design was achieved using the cDNA alignment tool followed by the “Specific” primer design option. The primers used can be found in Table S3.

## Electronic supplementary material

Below is the link to the electronic supplementary material.

Supplementary material 1 (XLSX 92 KB)

Supplementary material 2 (XLSX 62 KB)

Supplementary material 3 (XLSX 12 KB)

Supplementary material 4 (JPG 7719 KB)

Supplementary material 5 (JPG 736 KB)

## Abbreviations

bHLH: Basic helix-loop-helix
BLAST: Basic local alignment search tool
CEGMA: Core eukaryotic genes mapping approach
mRNA: Messenger ribonucleic acid
NGS: Next generation sequencing
oc: Orthology cluster
ORF: Open reading frame
PVP: Polyvinylpyrolidone
REAPR: Recognition of errors in assemblies using paired reads
rRNA: Ribosomal ribonucleic acid
TAIR: The Arabidopsis information resource
TPM: Transcripts per million
β-ME: β-mercaptoethanol

## References

[CR1] Bolger AM, Lohse M, Usadel B (2014). Trimmomatic: a flexible trimmer for Illumina sequence data. Bioinformatics.

[CR2] Buchfink B, Xie C, Huson DH (2015) Fast and sensitive protein alignment using DIAMOND. 12 59–60 10.1038/nmeth.3176 25402007

[CR3] Chang Z, Li G, Liu J, Zhang Y, Ashby C, Liu D, Cramer C, Huang X (2015). Bridger: a new framework for de novo transcriptome assembly using RNA-seq data. Genome Biol.

[CR4] Consortium TU (2015). UniProt: a hub for protein information. Nucleic Acids Res.

[CR5] Danisman S, van Dijk ADJ, Bimbo A, van der Wal F, Hennig L, de Folter S, Angenent GC, Immink RGH (2013). Analysis of functional redundancies within the Arabidopsis TCP transcription factor family. J Exp Bot.

[CR6] Deng X (2011). SeqGene: a comprehensive software solution for mining exome-and transcriptome-sequencing data. BMC Bioinformatics.

[CR7] Drummond A, Ashton B, Buxton S, Cheung M, Heled J, Kearse M, Moir R, Stones-Havas S, Sturrock S, Thierer T, Wilson A (2010) Geneious v5.0, http://www.geneious.com 10.1093/bioinformatics/bts199 PMC3371832 22543367

[CR8] Duangjit J, Bohanec B, Chan AP, Town CD, Havey MJ (2013). Transcriptome sequencing to produce SNP-based genetic maps of onion. Theor Appl Genet.

[CR9] Emms D, Kelly S (2015). OrthoFinder: solving fundamental biases in whole genome comparisons dramatically improves orthogroup inference accuracy. Genome Biol.

[CR10] Finn RD, Bateman A, Clements J, Coggill P, Eberhardt RY, Eddy SR, Heger A, Hetherington K, Holm L, Mistry J, Sonnhammer ELL, Tate J, Punta M (2014). Pfam: the protein families database. Nucleic Acids Res.

[CR11] Grabherr MG, Haas BJ, Yassour M, Levin JZ, Thompson DA, Amit I, Adiconis X, Fan L, Raychowdhury R, Zeng Q, Chen Z, Mauceli E, Hacohen N, Gnirke A, Rhind N, di Palma F, Birren BW, Nusbaum C, Lindblad-Toh K, Friedman N, Regev A (2011). Trinity: reconstructing a full-length transcriptome without a genome from RNA-Seq data. Nat Biotechnol.

[CR12] Haas BJ, Papanicolaou A, Yassour M, Grabherr M, Blood PD, Bowden J, Couger MB, Eccles D, Li B, Lieber M, MacManes MD, Ott M, Orvis J, Pochet N, Strozzi F, Weeks N, Westerman R, William T, Dewey CN, Henschel R, LeDuc RD, Friedman N, Regev A (2013). De novo transcript sequence reconstruction from RNA-Seq: reference generation and analysis with Trinity. Nat Protoc.

[CR13] Hou R, Bao Z, Wang S, Su H, Li Y, Du H, Hu J, Wang S, Hu X (2011). Transcriptome sequencing and de novo analysis for yesso scallop (Patinopecten yessoensis) Using 454 GS FLX. PLoS One.

[CR14] Hunt M, Kikuchi T, Sanders M, Newbold C, Berriman M, Otto T (2013). REAPR: a universal tool for genome assembly evaluation. Genome Biol.

[CR15] Huson DH, Auch AF, Qi J, Schuster SC (2007). MEGAN analysis of metagenomic data. Genome Res.

[CR39] International Rice Genome Sequencing Project (2005). The map-based sequence of the rice genome. Nature.

[CR17] Jones SI, Tan Y, Shamimuzzaman M, George S, Cunningham BT, Vodkin L (2015). Direct detection of transcription factors in cotyledons during seedling development using sensitive silicon-substrate photonic crystal protein arrays. Plant Physiol.

[CR18] Kamei CLA, Severing EI, Dechesne A, Furrer H, Dolstra O, Trindade LM (2016). Orphan crops browser: a bridge between model and orphan crops. Mol Breeding.

[CR19] Kamenetsky R, Okubo H (2013). Ornamental geophytes: from basic science to sustainable production.

[CR20] Kamenetsky R, Faigenboim A, Shemesh Mayer E, Ben Michael T, Gershberg C, Kimhi S, Esquira I, Rohkin Shalom S, Eshel D, Rabinowitch HD, Sherman A (2015). Integrated transcriptome catalogue and organ-specific profiling of gene expression in fertile garlic (Allium sativum L.). BMC Genomics.

[CR21] Khodorova N, Boitel-Conti M (2013). The role of temperature in the growth and flowering of geophytes. Plants.

[CR22] Lee J-Y, Colinas J, Wang JY, Mace D, Ohler U, Benfey PN (2006). Transcriptional and posttranscriptional regulation of transcription factor expression in Arabidopsis roots. Proc Natl Acad Sci USA.

[CR23] Li B, Dewey CN (2011). RSEM: accurate transcript quantification from RNA-Seq data with or without a reference genome. BMC Bioinformatics.

[CR24] Li Z, Trick HN (2005). Rapid method for high-quality RNA isolation from seed endosperm containing high levels of starch. Biotechniques.

[CR25] Li X, Wang C, Cheng J, Zhang J, da Silva JAT, Liu X, Duan X, Li T, Sun H (2014). Transcriptome analysis of carbohydrate metabolism during bulblet formation and development in Lilium davidii var. unicolor. BMC Plant Biol.

[CR26] Liu M, Qiao G, Jiang J, Yang H, Xie L, Xie J, Zhuo R (2012). Transcriptome sequencing and de novo analysis for ma bamboo (dendrocalamus latiflorus munro) Using the illumina Platform. PLoS One.

[CR27] Marguerat S, Bähler J (2010). RNA-seq: from technology to biology. CMLS Cell Mol Life Sci.

[CR28] Marinangeli PA, Hernandez LF, Pellegrini CP, Curvetto NR (2003). Bulblet differentiation after scale propagation of Lilium longiflorum. J Am Soc Hortic Sci.

[CR29] Martín-Trillo M, Cubas P (2010). TCP genes: a family snapshot 10years later. Trends Plant Sci.

[CR30] Ming R, VanBuren R, Wai CM, Tang H, Schatz MC, Bowers JE, Lyons E, Wang M-L, Chen J, Biggers E, Zhang J, Huang L, Zhang L, Miao W, Zhang J, Ye Z, Miao C, Lin Z, Wang H, Zhou H, Yim WC, Priest HD, Zheng C, Woodhouse M, Edger PP, Guyot R, Guo H-B, Guo H, Zheng G, Singh R, Sharma A, Min X, Zheng Y, Lee H, Gurtowski J, Sedlazeck FJ, Harkess A, McKain MR, Liao Z, Fang J, Liu J, Zhang X, Zhang Q, Hu W, Qin Y, Wang K, Chen L-Y, Shirley N, Lin Y-R, Liu L-Y, Hernandez AG, Wright CL, Bulone V, Tuskan GA, Heath K, Zee F, Moore PH, Sunkar R, Leebens-Mack JH, Mockler T, Bennetzen JL, Freeling M, Sankoff D, Paterson AH, Zhu X, Yang X, Smith JAC, Cushman JC, Paull RE, Yu Q (2015). The pineapple genome and the evolution of CAM photosynthesis. Nat Genet.

[CR31] Mondragón-Palomino M, Trontin C (2011). High time for a roll call: gene duplication and phylogenetic relationships of TCP-like genes in monocots. Ann Bot (Lond).

[CR32] Narita NN, Moore S, Horiguchi G, Kubo M, Demura T, Fukuda H, Goodrich J, Tsukaya H (2004). Overexpression of a novel small peptide ROTUNDIFOLIA4 decreases cell proliferation and alters leaf shape in Arabidopsis thaliana. Plant J.

[CR33] Neale D, Wegrzyn J, Stevens K, Zimin A, Puiu D, Crepeau M, Cardeno C, Koriabine M, Holtz-Morris A, Liechty J, Martinez-Garcia P, Vasquez-Gross H, Lin B, Zieve J, Dougherty W, Fuentes-Soriano S, Wu L-S, Gilbert D, Marcais G, Roberts M, Holt C, Yandell M, Davis J, Smith K, Dean J, Lorenz W, Whetten R, Sederoff R, Wheeler N, McGuire P, Main D, Loopstra C, Mockaitis K, deJong P, Yorke J, Salzberg S, Langley C (2014). Decoding the massive genome of loblolly pine using haploid DNA and novel assembly strategies. Genome Biol.

[CR34] Nicolas M, Rodríguez-Buey María L, Franco-Zorrilla José M, Cubas P (2015). A recently evolved alternative splice site in the BRANCHED1a gene controls potato plant architecture. Curr Biol.

[CR35] Parra G, Bradnam K, Korf I (2007). CEGMA: a pipeline to accurately annotate core genes in eukaryotic genomes. Bioinformatics.

[CR36] Riechmann JL, Heard J, Martin G, Reuber L, Jiang C-Z, Keddie J, Adam L, Pineda O, Ratcliffe OJ, Samaha RR, Creelman R, Pilgrim M, Broun P, Zhang JZ, Ghandehari D, Sherman BK, Yu G -L. (2000). Arabidopsis transcription factors: genome-wide comparative analysis among eukaryotes. Science.

[CR37] Riesgo A, Andrade SCS, Sharma PP, Novo M, Pérez-Porro AR, Vahtera V, González VL, Kawauchi GY, Giribet G (2012). Comparative description of ten transcriptomes of newly sequenced invertebrates and efficiency estimation of genomic sampling in non-model taxa. Front Zool.

[CR38] Schatz MC, Delcher AL, Salzberg SL (2010). Assembly of large genomes using second-generation sequencing. Genome Res.

[CR40] Shahin A, van Kaauwen M, Esselink D, Bargsten JW, van Tuyl JM, Visser RGF, Arens P (2012). Generation and analysis of expressed sequence tags in the extreme large genomes Lilium and Tulipa. BMC Genomics.

[CR16] The Arabidopsis Genome Initiative (2000). Analysis of the genome sequence of the flowering plant Arabidopsis thaliana. Nature.

[CR41] Treangen TJ, Salzberg SL (2011). Repetitive DNA and next-generation sequencing: computational challenges and solutions. Nat Rev Genet.

[CR42] Tuskan GA, DiFazio S, Jansson S, Bohlmann J, Grigoriev I, Hellsten U, Putnam N, Ralph S, Rombauts S, Salamov A, Schein J, Sterck L, Aerts A, Bhalerao RR, Bhalerao RP, Blaudez D, Boerjan W, Brun A, Brunner A, Busov V, Campbell M, Carlson J, Chalot M, Chapman J, Chen GL, Cooper D, Coutinho PM, Couturier J, Covert S, Cronk Q, Cunningham R, Davis J, Degroeve S, xe, jardin A, dePamphilis C, Detter J, Dirks B, Dubchak I, Duplessis S, Ehlting J, Ellis B, Gendler K, Goodstein D, Gribskov M, Grimwood J, Groover A, Gunter L, Hamberger B, Heinze B, Helariutta Y, Henrissat B, Holligan D, Holt R, Huang W, Islam-Faridi N, Jones S, Jones-Rhoades M, Jorgensen R, Joshi C, Kangasj, xe, rvi J, Karlsson J, Kelleher C, Kirkpatrick R, Kirst M, Kohler A, Kalluri U, Larimer F, Leebens-Mack J, Lepl CJ, Locascio P, Lou Y, Lucas S, Martin F, Montanini B, Napoli C, Nelson DR, Nelson C, Nieminen K, Nilsson O, Pereda V, Peter G, Philippe R, Pilate G, Poliakov A, Razumovskaya J, Richardson P, Rinaldi C, Ritland K, Rouz, xe, P., Ryaboy D, Schmutz J, Schrader J, Segerman B, et al (2006) The Genome of black cottonwood, populus trichocarpa (Torr. & Gray). Science 313:1596–1604 10.1126/science.1128691 16973872

[CR43] Villacorta-Martin C, Núñez de Cáceres González FF, de Haan J, Huijben K, Passarinho P, Lugassi-Ben Hamo M, Zaccai M (2015). Whole transcriptome profiling of the vernalization process in Lilium longiflorum (cultivar White Heaven) bulbs. BMC Genomics.

[CR44] Wang G, Fiers M (2010). CLE peptide signaling during plant development. Protoplasma.

[CR45] Wang J, Yang Y, Liu X, Huang J, Wang Q, Gu J, Lu Y (2014). Transcriptome profiling of the cold response and signaling pathways in Lilium lancifolium. BMC Genomics.

[CR46] Zhang H, Jin J, Tang L, Zhao Y, Gu X, Gao G, Luo J (2011). PlantTFDB 2.0: update and improvement of the comprehensive plant transcription factor database. Nucleic Acids Res.

