# Supplementary figures and images for: Elucidating and mining the Tulipa and Lilium transcriptomes
Source: Plant Mol Biol. 2016 Jul 7;92(3):249–61. doi: 10.1007/s11103-016-0508-1 (PMC5566170; doi:10.1007/s11103-016-0508-1)

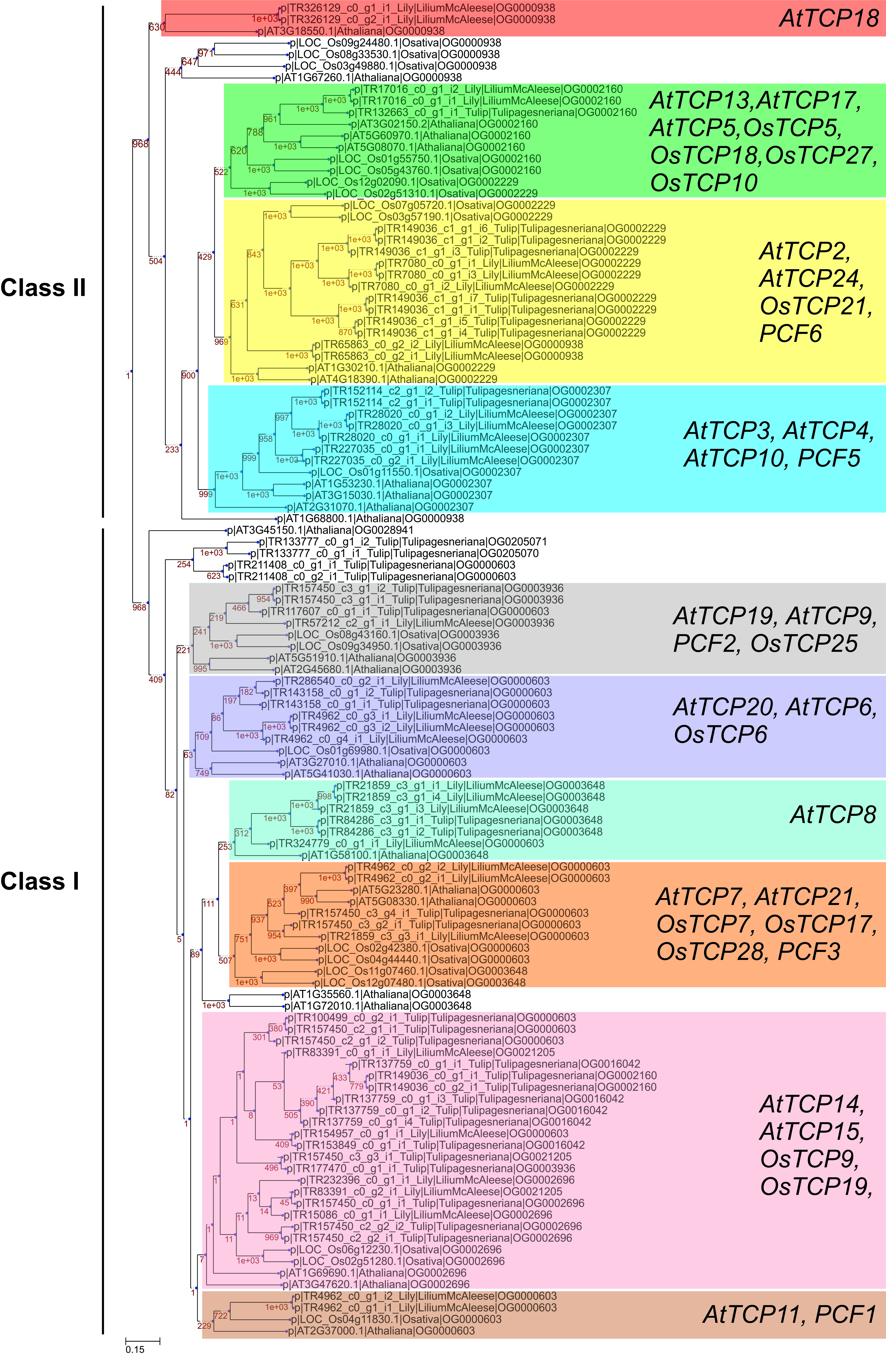

Supplement: Supplementary file 4 — Supplementary material 4 (JPG 7719 KB) [file 11103_2016_508_MOESM4_ESM.jpg]

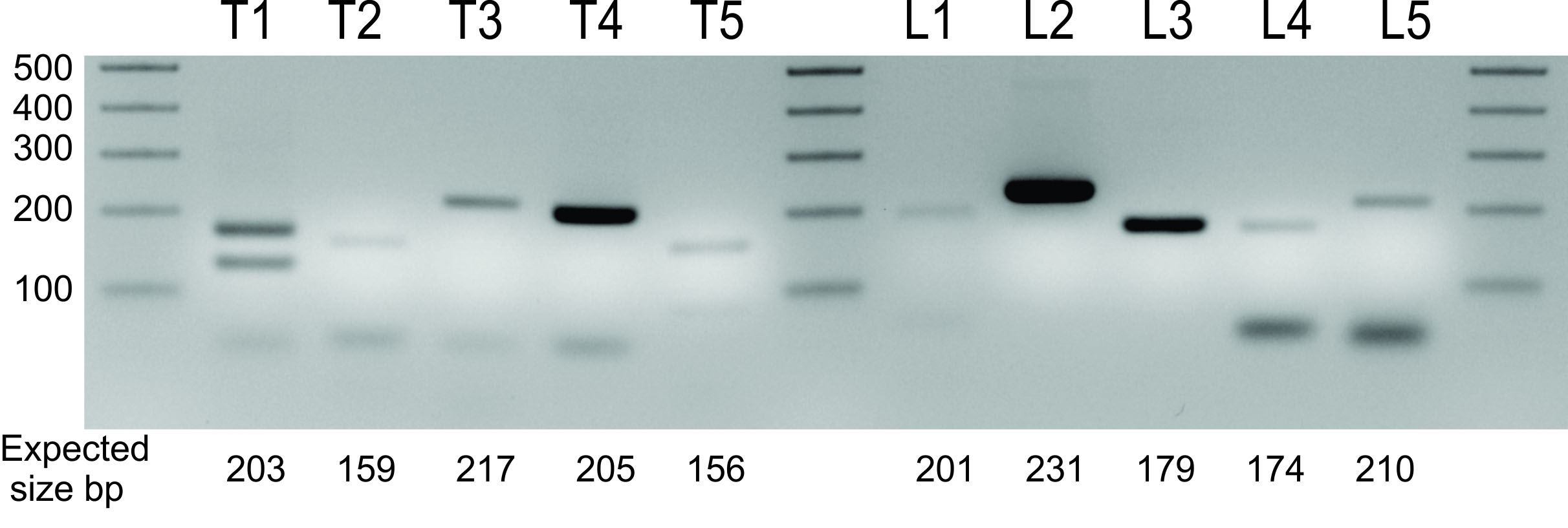

Supplement: Supplementary file 5 — Supplementary material 5 (JPG 736 KB) [file 11103_2016_508_MOESM5_ESM.jpg]
